# Conjunctivitis outbreak associated with Enterovirus Type C in Luzira Prisons, Uganda, February-April 2024

**DOI:** 10.1016/j.ijregi.2026.100929

**Published:** 2026-05-29

**Authors:** Hannington Katumba, Richard Migisha, Charity Mutesi, Emmanuel Mfitundinda, Joanita Nalwanga, Joyce Owens Kobusingye, Daniel Wenani, Loryndah Olive Namakula, Emmanuel Okello Okiror, Janet Lubega Kobusinge, Gertrude Abbo, Annet Mary Namusisi, Bridget Ainembabazi, Patrick Kwizera, Wilfred Opeli, Winnie Agwang, Esther Nabatta, Tracy Maureen Rutogire, Ritah Namusoosa, Samuel Lugwana, Lilian Bulage, Benon Kwesiga, Samuel Gidudu, Alex Riolexus Ario

**Affiliations:** Uganda Public Health Fellowship Program, Uganda National Institute of Public Health, Kampala, Uganda

**Keywords:** Conjunctivitis, Keratoconjunctivitis, Outbreaks, Prison, Uganda

## Abstract

**Objectives:**

On March 7, 2024, the Ministry of Health was notified of a rising number of inmates with suspected conjunctivitis in Luzira Prisons in Uganda. We investigated this outbreak to determine its cause and extent, identify risk factors, and recommend evidence-based control measures.

**Methods:**

We investigated the outbreak in four prisons: Kampala Remand Prison, Murchison Bay Prison, Luzira Upper Prison, and Luzira Women Prison. A suspected case was defined as the onset of redness in one or both eyes with more than or equal to one of the following: tearing, discharge, grainy sensation, itching, pain, or swelling, in any Luzira Prison resident from February 1 to April 3, 2024. We identified cases using medical records and active case searches among prisoners. We administered structured questionnaires and collected conjunctival swabs for polymerase chain reaction testing. We assessed prisoner movement, handwashing, isolation practices, and administration of eye medications. We compared exposures of 200 randomly selected cases and 200 controls in a case-control study. Using logistic regression, we conducted multivariable analysis to identify risk factors.

**Results:**

We recorded 1935 cases; the overall attack rate was 23% (1935/8518), varying by prison: Murchison Bay Prison (41%; 1229/3000), Kampala Remand Prison (33%; 610/1835), Luzira Women Prison (12%; 83/670), and Luzira Upper Prison (0.4%; 13/3013). With no associated deaths, most cases resolved within 4-5 days. Of the 10 samples tested, 4 (40%) were positive for Enterovirus Type C. Sharing of eye medication (adjusted odds ratio: 5.2, 95% confidence interval: 2.0-6.6), and infrequent handwashing increased the odds of infection (adjusted odds ratio: 5.8, 95% confidence interval: 3.5-9.6). Prisoner mixing took place during a plea-bargain session amid the outbreak. Symptomatic patients were isolated for 3 days.

**Conclusion:**

The outbreak was associated with Enterovirus Type C and was facilitated by prisoner mixing, sharing of eye medications, short isolation periods, and inadequate hand hygiene practices. Improving infection prevention and control measures, including strict isolation, individualized eye medication, and enhanced hand hygiene practices, could prevent future outbreaks in similar settings.

## Highlights

• Enterovirus Type C caused a conjunctivitis outbreak affecting 23% of prison inmates.
• Sharing eye medication and infrequent handwashing were the strongest risk factors.
• Prisoner mixing and short isolation periods accelerated the spread across prison units.
• We present actionable infection prevention and control (IPC) evidence for closed settings to mitigate future outbreaks.

## Introduction

Conjunctivitis, characterized by inflammation of the ocular mucous membranes, arises from diverse etiologies, including viral, bacterial, allergic, parasitic, and non-specific causes [1,2]. Viruses account for approximately 80% of acute cases, with outbreaks predominantly associated with adenoviruses and enteroviruses, particularly Enterovirus Type D and Enterovirus Type C, the causative agents of acute hemorrhagic conjunctivitis [3]. The disease has a short incubation period of 12 to 48 hours [4]. Acute hemorrhagic conjunctivitis is highly contagious, spreading rapidly among persons through direct contact or indirectly through the sharing of bedding, clothing, eyeglasses, and eye medications. Outbreaks are more common in crowded settings such as schools, military barracks, prisons, and refugee camps [5,6].

In Uganda, the first reported outbreak of conjunctivitis occurred in 2010 in 26 districts, followed by another outbreak in Gulu District in November 2016 among inmates in a prison. Both outbreaks were caused by Coxsackievirus CVA24 [[7], [8], [9]]. Other viruses associated with these outbreaks include Enterovirus-70 and human adenovirus species [[10], [11], [12], [13]]. The rapid transmission of these viruses is further facilitated by factors such as elevated temperatures, overcrowding, and high humidity [6,13].

In February 2024, prisoners with conjunctivitis were remanded to Kampala Remand Prison in Luzira after being transferred from a police post within Kampala City. The Kampala Capital City Authority notified the Ministry of Health (MoH) on March 7, 2024, about a rising number of conjunctivitis cases (n = 314) within Luzira Prisons. We conducted an investigation to identify the cause and scope of the outbreak, assess the risk factors contributing to its spread, and recommend evidence-based measures to control the outbreak and prevent similar outbreaks in the future.

## Methods

### Outbreak setting

The outbreak occurred in Luzira Prisons, located in Nakawa Division, Kampala, Uganda. It is a prison for both males and females. It is a complex of four different prisons in the same geographical location: Luzira Women Prison (LWP), Murchison Bay Prison (MBP), Luzira Upper Prison (LUP), and Kampala Remand Prison (KRP). Initially designed to accommodate 1700 inmates, the prison had a population of nearly 8000 inmates at the time of the investigation. Each of the four prisons has a Health Center III-level health facility.

MBP is unique in that it houses the referral hospital (Murchison Bay Hospital) for all Uganda prisons; only sick inmates are admitted there. The referral hospital had a dedicated ophthalmic clinical officer, although the ophthalmic clinic lacked basic diagnostic tools. The prison had approximately 3000 inmates despite its intended capacity of only 600. Additionally, it had a designated isolation block, which was overcrowded.

KRP has frequent prisoner traffic. Daily, about 250 inmates leave the prison to attend court sessions, some inmates are released by the court, and new crime suspects are remanded. It was designed for 600 inmates; however, it housed about 1840 inmates during this outbreak. The prisoners sleep in wards, equivalent to dormitories. The prison has 12 wards and houses inmates serving short-term sentences, ranging from one day to 19 years.

In contrast, LUP is a maximum-security prison and has much less frequent inflows of new inmates, as it houses only long-term convicts serving 20 years or more. By the time of this investigation, the prison had 3013 inmates. Luzira Women’s Prison is Uganda’s maximum-security prison for female inmates, with less frequent inflows of new inmates and accommodating 670 inmates at the time of the investigation.

At the time of this outbreak in Luzira Prisons, the Uganda MoH had received reports of conjunctivitis in communities in Kampala City.

### Case definition and case finding

We defined a suspected case as the onset of redness in one or both eyes with one or more of the following: tearing, discharge, grainy sensation, itching, pain, or swelling, in a resident of Luzira Prison from February 1, 2024, to April 3, 2024. A confirmed case was a suspected case with a positive laboratory result. We systematically identified cases by reviewing medical records at the health facilities located within the respective prisons in Luzira. We also actively searched for cases among inmates with the help of health workers and the “doctor wards” and generated a line list. A “doctor ward,” a term routinely used in this prison, was a fellow inmate informally selected from each respective ward to perform roles equivalent to those of community health workers.

### Laboratory investigations

Conjunctival swabs were collected from 10 suspected cases for polymerase chain reaction testing and gene sequencing at the Uganda Virus Research Institute, a national reference laboratory for research and surveillance.

### Environmental assessment

We assessed potential factors contributing to the introduction and spread of conjunctivitis within the prisons. These included prisoner movement, interactions between inmates from different facilities, and the availability and functionality of handwashing facilities. We assessed how patients with conjunctivitis were managed by interviewing affected patients, ‘doctor wards,’ and health facility workers. We also assessed the administration of eye medication and the isolation of identified patients.

### Descriptive epidemiology

We computed attack rates (ARs), stratified by prison, based on the population at the time of the investigation. The population denominators for each prison were based on estimates provided by the respective prison administrations at the start of the investigation (March 12, 2024). These estimates reflected the best available counts on that date and were used as denominators for calculating ARs because inmate numbers fluctuated daily due to admissions, releases, and court appearances. We described cases by clinical manifestations and likely exposures and constructed an epidemic curve to show the distribution of cases over time.

### Hypothesis generation

We generated hypotheses from the descriptive epidemiology and hypothesis-generating discussions with ‘doctor wards,’ health facility staff, and prison officers in charge of welfare and reception about potential risk factors for the transmission of conjunctivitis. The interviews included questions on prisoner flow, sleeping next to someone who was sick, movement outside the current prison, isolation of infected inmates, availability of handwashing facilities, administration of eye medication, and receiving visitors.

### Case-control study

A prison-based case-control study was conducted. Inmates presenting with symptoms typical of conjunctivitis from KRP and MBP were enrolled in the study because these were the most affected prisons. After treatment was administered, inmates who were willing and able to provide consent were invited to participate in the interview. Verbal consent was obtained from participants after explaining the aims and procedures of the study. The questionnaire was developed in English (Uganda’s official language) and administered by field epidemiologists from the Uganda National Institute of Public Health, all of whom were fluent in English and Luganda. At the start of each interview, participants chose either English or Luganda, and none lacked proficiency in at least one of these languages.

Controls were selected from the same prisons as the cases. For each case, one control was selected (i.e. a 1:1 ratio). The outcome of interest in our study was conjunctivitis, and the primary exposure for cases and controls was contact with a patient with conjunctivitis. Other exposures included WASH habits, such as washing hands with soap and water, frequency of handwashing, sharing eye ointment, and sharing personal items such as blankets, sponges, and uniforms.

The sample size was calculated using Fleiss’s method for unmatched case-control studies. Assuming a control exposure prevalence (P₀) of 51%—based on a prior conjunctivitis survey among Luzira inmates—a 1:1 case-control ratio, 80% power, and a two‐sided α of 0.05, we determined that 200 cases and 200 controls were required. Data were collected using interviewer-administered questionnaires that were electronically uploaded to the Kobo Toolbox. We identified risk factors associated with infection using multivariable logistic regression. Independent variables with a *P*-value < 0.05 were used to provide adjusted odds ratios with 95% confidence intervals (CIs), as well as variables considered important based on the literature. Backward elimination was used to include variables in the final model. The most parsimonious model was selected based on the lowest Akaike Information Criterion and Bayesian Information Criterion values.

## Results

### Descriptive epidemiology

**Figure 1:**
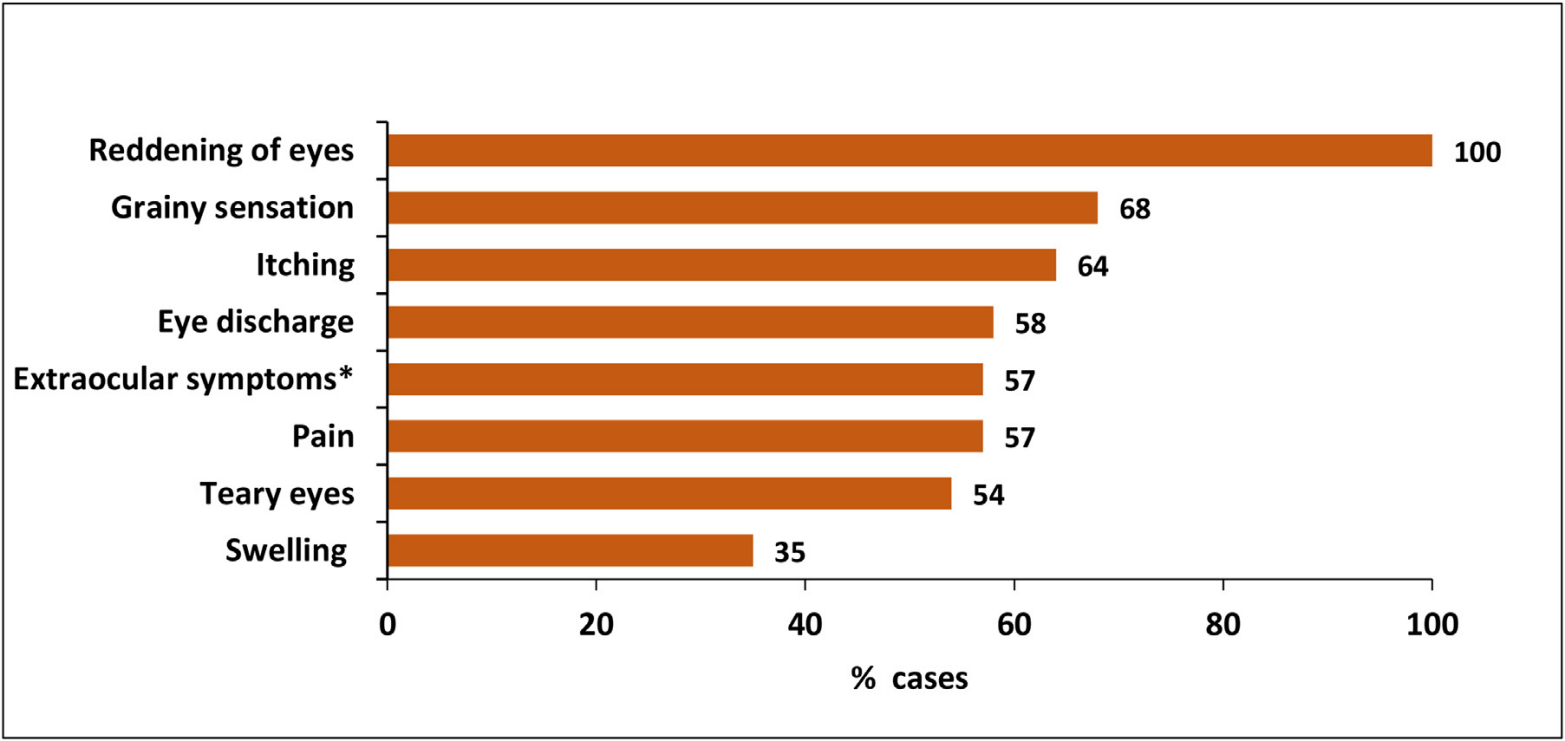
Distribution of symptoms among patients during a conjunctivitis outbreak, Luzira Prison, February-March 2024 (n = 1935). *Extraocular symptoms included fever, headache, cough, flu, general body weakness, runny nose, and dizziness.

A total of 1935 patients with conjunctivitis were identified in Luzira Prisons by April 2, 2024, with no deaths reported. Of these, four tested positive for Enterovirus Type C. The mean age of patients was 30 years (SD = 9.4), and most recovered within 4-5 days. All patients reported presenting with reddening of the eyes (Figure 1).

The overall AR was 23% (1935/8518), with males being more affected (96%, n=1852) than females (4%, n = 83). MBP, with 1229 cases (AR: 41/100), was the most affected prison (Table 1).

**Table 1:** ARs by prison during an outbreak of conjunctivitis, Luzira Prison, Kampala, Uganda, February 1- April 2, 2024.

AR by prison			
Prison name	Cases	Total population^a^	AR (AR/100)
Murchison Bay Prison	1229	3000	41
Kampala Remand Prison	610	1835	33
Luzira Women's Prison^b^	83	670	12
Luzira Upper Prison	13	3013	0.4
Total	1935	8518	23

AR by sex			

Sex	Cases	Total population	AR (AR/100)

Male	1852	7848	24
Female	83	670	12
**Total**	**1935**	**8518**	**23**

Abbreviation: AR, attack rate.

a Population estimates provided by prison authorities on March 12, 2024.

b This is an All-Female prison.

**Figure 2:**
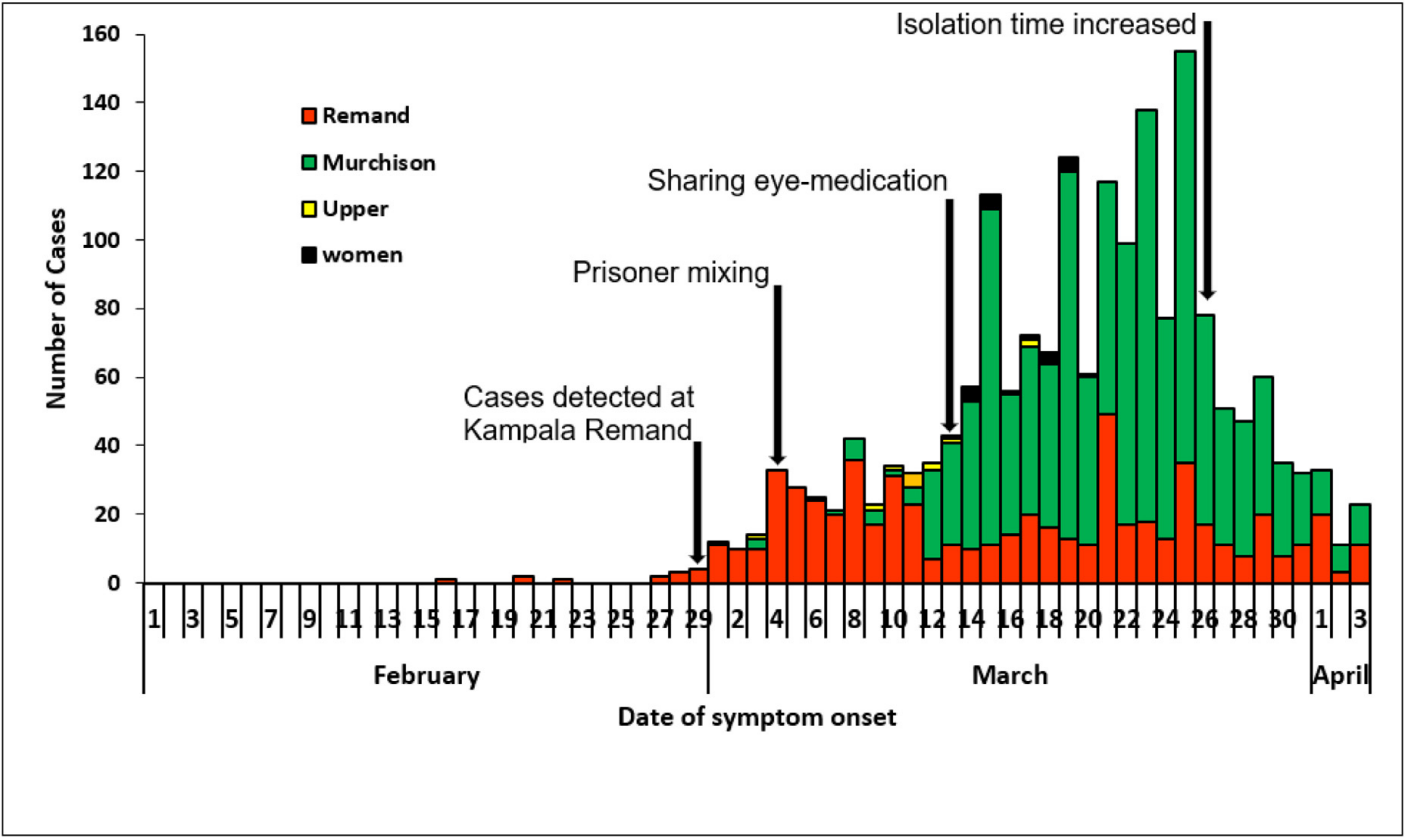
Distribution of conjunctivitis cases over time among patients in Luzira Prisons, February 1-April 3, 2024 (n = 1935).

This outbreak started with the admission of infected inmates to KRP in February 2024. Sharing of topical eye treatments between infected and uninfected inmates started on March 12, 2024. On March 15, there was a peak in the number of resident cases. This sharing of eye medication was discontinued on March 19, 2024, and inmates’ medication was administered separately by the “doctor ward.” Immediate isolation protocols for newly admitted inmates were initiated thereafter. Infected inmates continued to be admitted into KRP beyond the time of the investigation (Figure 2).

Some cases reported in MBP involved inmates referred to the prison hospital. These patients were promptly isolated upon admission. On March 5, 2024, prisoner mixing occurred during a plea-bargain meeting held within KRP, which was attended by some prisoners from Murchison Bay and Luzira Upper Prison (Figure 2).

Subsequently, more cases of conjunctivitis emerged among inmates at MBP beginning on March 6, 2024. As a preventive measure, tetracycline eye ointment (TEO) was distributed two days later. TEO served both as prophylaxis for those without symptoms and as a treatment for affected individuals. Despite these interventions, the number of cases steadily increased over time, with multiple peaks in incidence (Figure 2).

Starting on March 26, additional public health measures were implemented. These included prompt isolation of identified cases for at least five days, providing Continuous Medical Education sessions for healthcare workers and laboratory staff, and conducting health talks for inmates. These public health measures preceded a noticeable decline in the number of reported cases (Figure 2).

### Luzira Women’s Prison

Cases were identified in March 2024 among inmates who attended court sessions and reported sharing a bus with an infected inmate from Murchison Bay on their way to and from court (Figure 2).

### Luzira Upper Prison

The data obtained from LUP indicated that cases of conjunctivitis were routinely recorded in the health facility records.

Overall, the outbreak in MBP was preceded by that in KRP, and by April 3, a total of 1935 suspected cases had been reported to the prison health facilities from the four prisons.

### Laboratory findings

From the 10 samples collected and submitted for polymerase chain reaction testing, four (40%) were positive for Enterovirus Type C.

### Environmental and case management assessment findings

KRP transports 150-200 inmates every day to attend different court sessions. In contrast, the maximum-security prison (LUP) transports an average of 80 inmates to court daily, and these courts are usually different from those attended by inmates from both KRP and MBP.

Some inmates were released by the court, while, at the same time, new inmates were remanded. MBP receives prisoners referred from other prisons for medical attention at MBP Hospital. There was prisoner mixing during a plea-bargain session at KRP, where some inmates from MBP and LUP attended.

We observed the presence of tap and tank water with soap at the prison main gate and at the entrances to seven of the 12 prison wards in KRP and 16 of the 17 wards in MBP. All the wards had indoor handwashing points, with the water pre-mixed with liquid soap.

In the earlier days of the outbreak, patients with conjunctivitis were isolated and treated. Later, the number of cases was so high that isolation was no longer possible. Identified patients were treated in the prison wards where they resided. Treatment was administered topically by the “doctor ward” using their hands for all inmates, regardless of disease status. This was not observed in either the LUP or the LWP. By this time, only those with severe eye discharge were isolated. Isolation initially lasted only up to 3 days in MBP. However, in LUP, isolation was sustained until prisoners fully recovered from the infection.

### Hypothesis generation findings

Based on the descriptive epidemiology, laboratory, and environmental assessment findings, we hypothesized that sleeping next to someone who was sick and sharing eye medication was associated with propagation of the outbreak among inmates. Poor hand hygiene was associated with increased transmission of conjunctivitis among inmates (Table 2).

**Table 2:** Exposures among 200 cases during a conjunctivitis outbreak, Luzira Prisons, Uganda, March-April 3, 2024.

Factor	Cases (n = 200)	%
Washed face daily	195	98
Slept next to someone sick	175	88
Washed hands with soap	139	70
Moved out of prison	114	57
Shared blankets	63	32
Shared eye medication	51	26
Received visitors	34	17

### Case-control findings

We enrolled 200 controls for the case-control study. At the bivariate level, sharing eye medication (crude odds ratio [cOR]: 3.6, 95% CI = 2.0-6.6), sleeping next to someone who was sick (cOR: 2.2, 95% CI = 1.3-3.7), and reduced handwashing frequency (cOR: 5.2, 95% CI = 3.2-8.3) were associated with conjunctivitis. Results from multivariable analysis indicated that sharing eye medication (aOR: 5.2, 95% CI = 2.9-10.5, *P* < 0.001) and reduced frequency of handwashing (aOR: 12.8, 95% CI = 5.7-28.7, *P* < 0.001) were significantly associated with acquiring the infection among inmates (Table 3).

**Table 3:** Factors associated with conjunctivitis among case-persons during an outbreak, Luzira Prisons, Uganda, March-April 3, 2024.

	Cases		Controls		Bivariate		Multivariable		
Variable	n = 200	%	n = 200	%	cOR	95% CI	aOR	95% CI	*P*-value
**Sharing eye medication**									
No	149	75	183	91	Ref	Ref	Ref	Ref	
Yes	51	25	17	9	3.6	2.0-6.6	5.2	2.9-10.5	<0.001
**Sleeping next to someone sick**									
No	26	13	49	25	Ref	Ref	Ref	Ref	
Yes	175	87	150	75	2.2	1.3-3.7	2.3	1.2-4.2	0.0064
**Received visitors**									
No	167	83	131	66	Ref	Ref			
Yes	34	17	68	34	0.4	0.2-0.6			
**Moved out of prison**									
No	87	43	69	35	Ref	Ref			
Yes	114	57	130	65	0.7	0.5-1.1			
**Shared blankets**									
No	138	69	112	56	Ref	Ref			
Yes	63	31	87	44	0.6	0.4-0.9			
**Did not wash hands with soap and water**									
Yes	62	31	8	4	Ref	Ref	Ref	Ref	
No	139	69	191	96	10.6	4.9-22.9	12.9	5.7-28.7	<0.001
**Frequency of washing hands**									
Frequently/always	105	48	169	85	Ref	Ref	Ref	Ref	
Rarely/ occasionally	96	52	30	15	5.2	3.2-8.3	5.8	3.5-9.6	<0.001
									

Abbreviations: aOR, adjusted odds ratio; cOR, crude odds ratio; Ref, reference category.

## Discussion

We investigated an outbreak of conjunctivitis within a Ugandan prison setting to assess the magnitude of the outbreak, identify associated risk factors, and recommend control measures. The outbreak primarily affected KRP and MBP, where the ARs were notably higher than those in LUP and LWP. Most cases were mild, with rapid resolution within 4-5 days, and no fatalities were reported. Laboratory testing identified Enterovirus Type C as the likely cause. Risk factors, including sharing eye medication and infrequent handwashing, were strongly associated with increased infection. Interactions between inmates from different prisons and suboptimal isolation of symptomatic individuals likely contributed to the spread. These findings highlight the need for improved hygiene practices and stronger isolation protocols in high-density, closed environments such as prisons, along with consideration of structural expansion.

The conjunctivitis outbreak was likely caused by Enterovirus Type C, supported by the clinical presentation, which was consistent with viral conjunctivitis, and laboratory confirmation in a subset of cases (40% of tested samples). This aligns with findings from other outbreak investigations, indicating that enteroviruses are the primary cause of most viral conjunctivitis outbreaks [[1], [2], [3],^7^,[14], [15], [16]].

MBP was the most affected prison. The variation in ARs can be attributed to the unique operational characteristics of each prison. For example, KRP experiences a high turnover of inmates, with new arrivals from police cells and courts, which likely contributed to the initial case and subsequent spread of conjunctivitis. The outbreak at MBP followed that at KRP, likely due to prisoner mixing. Inmates from MBP who attended a plea-bargain session at KRP were among the first cases. These meetings, which bring together inmates from different prison units, create a conducive environment for disease transmission during outbreaks of infectious diseases. To prevent such scenarios, prison authorities should consider organizing separate plea-bargain sessions for different prisons to help reduce the risk of cross-prison transmission [10].

The administration of topical eye medications may have inadvertently contributed to the propagation of the outbreak among inmates. Prison ward leaders, referred to as ‘doctor wards,’ were administering eye medications to both infected and uninfected inmates using the same tubes and initially applying TEO directly with their bare hands. This direct contact likely facilitated transmission of the infection from infected to uninfected inmates. Previous studies have shown that sharing eye medications can promote the spread of conjunctivitis, with additional evidence highlighting an increased risk associated with such practices [15]. To mitigate the rapid spread of infection within prison settings, it is essential to reduce contact between infected and uninfected inmates. Implementing screening protocols, ensuring prompt isolation, and providing individualized treatment for inmates with conjunctivitis could significantly help control outbreaks.

Initially, all identified patients were isolated; however, as the number of cases increased significantly, isolating every affected individual became impractical due to space constraints. The prison was already operating at approximately 300% of its original capacity. As a result, isolation was limited to individuals with eye discharge and lasted for a maximum of 3 days. Previous studies have demonstrated that isolation of infected individuals is an effective control measure [17]. Based on our findings, we recommend extending the isolation period to the recommended 5 days during future outbreaks to reduce transmission risk among inmates. Additionally, the government could consider decongesting prisons by constructing additional prison wards, thus providing more space for inmates.

After the isolation period, symptomatic inmates were returned to their respective wards, and the remaining patients were managed within overcrowded conditions, leading to inevitable close contact between infected and uninfected individuals. Proximity to infected inmates was associated with higher odds of developing conjunctivitis. This finding aligns with studies from Brazil, the USA, and China, where conjunctivitis spread through proximity [5].

In this outbreak, sharing fomites (blankets) among inmates was associated with lower odds of infection. This should be interpreted cautiously, as many symptomatic individuals were initially isolated from others to reduce transmission, making them less likely to share blankets and other personal effects. Similarly, receiving visitors was also associated with lower odds of developing conjunctivitis, likely because these individuals were isolated, while asymptomatic individuals continued to receive and see visitors. Moreover, sharing blankets and receiving visitors were self-reported. Such responses could have been affected by social desirability bias, with respondents likely perceiving such behaviors/actions as factors in disease transmission.

Frequent handwashing with soap and water prevented infection spread, consistent with findings from previous studies elsewhere [14]. During conjunctivitis outbreaks, prison authorities should prioritize the availability of handwashing facilities to promote better hygiene practices and reduce the risk of transmission.

Early case confirmation and prompt initiation of control measures are critical to limiting transmission, particularly in overcrowded settings. Future responses should prioritize rapid case verification, laboratory confirmation, risk assessment, and timely implementation of infection prevention and control actions.

Overall, this outbreak highlighted the vulnerability of overcrowded prison environments to rapid disease transmission and underscored the need to strengthen infection prevention and control measures. Early identification and isolation of symptomatic inmates, improved access to handwashing facilities and soap, and strict hygiene during eye treatment are critical. The outbreak revealed that ‘doctor wards,’ who are peer health workers, were instrumental in the transmission of the disease through hand application of eye medication, emphasizing the urgent need for continuous infection prevention and control training for this and other infectious disease outbreaks. Early case confirmation and prompt initiation of control measures are critical to limiting transmission, particularly in overcrowded settings.

### Study limitations

A key limitation of this study is its scope, as we focused exclusively on the outbreak within Luzira Prisons, which limited our ability to assess the broader evolution and extent of the outbreak across other prisons in Uganda. As a result, potential clusters in other facilities or communities, such as schools, were not explored. This restriction prevented us from determining whether these clusters were epidemiologically linked to the Luzira Prison outbreak. Additionally, laboratory confirmation was conducted on a limited number of samples, which may have underestimated the true prevalence of Enterovirus Type C and limits the certainty of attributing the entire outbreak to this virus. Furthermore, as this investigation was conducted as a public health emergency response, detailed molecular characterization data beyond confirmation of Enterovirus Type C were not formally documented or relayed to the field investigation team. The lack of these broader epidemiological and granular laboratory insights limited our ability to develop more comprehensive, evidence-based control measures.

## Conclusion

Our investigation identified Enterovirus Type C as the causative agent of the conjunctivitis outbreak, supported by laboratory confirmation in a subset of cases and the clinical presentation. The outbreak had varying impacts across different prison facilities. The initial transmission was linked to the admission of an infected inmate, with further spread attributed to inmate interactions during a mass meeting (plea-bargain sessions). Contributing risk factors for transmission included sharing topical eye medications, overcrowding, close contact between symptomatic and asymptomatic inmates, and inadequate isolation practices. However, frequent handwashing was associated with a reduced risk of infection. We recommend strengthening infection prevention and control measures, including strict isolation of affected individuals, individualized eye medication, and enhanced hand hygiene practices to mitigate the risk of future outbreaks. Consideration should be given to structural expansion of prison facilities to reduce inmate overcrowding.

## Public health actions

During the investigation, we conducted sensitization sessions for inmates, emphasizing the importance of proper hand hygiene. We also trained the "doctor ward" staff on the correct administration of eye ointments and drops, highlighting the need for prompt reporting and referral of suspected cases to health facilities. Additionally, continuing medical education sessions were held for healthcare workers, and clinical and laboratory staff received training on sample collection, storage, and referral procedures. These activities were all based on evidence-based management strategies from other studies. In collaboration with the prison administration and health facility staff, we implemented immediate control measures. All identified patients during case finding were referred to the prison health facility for further management. Following the dissemination of our findings, the isolation period was extended from 3 days to 5-7 days [18].

We conducted an assessment of the prison laboratory’s outbreak readiness and provided recommendations for improvement to the prison authorities and relevant partners. A preliminary report of the findings was presented to the Public Health Commission of Uganda Prison Services for further review and action.

## CRediT authorship contribution statement

**Hannington Katumba:** Formal analysis, Investigation, Methodology, Project administration, Visualization, Writing – original draft, Writing – review & editing. **Richard Migisha:** Conceptualization, Investigation, Supervision, Writing – review & editing. **Charity Mutesi:** Formal analysis, Investigation, Writing – original draft, Writing – review & editing. **Emmanuel Mfitundinda:** Formal analysis, Investigation, Writing – original draft, Writing – review & editing. **Joanita Nalwanga:** Investigation, Writing – original draft, Writing – review & editing. **Joyce Owens Kobusingye:** Investigation, Writing – original draft, Writing – review & editing. **Daniel Wenani:** Investigation, Writing – original draft, Writing – review & editing. **Loryndah Olive Namakula:** Investigation, Writing – original draft, Writing – review & editing. **Emmanuel Okello Okiror:** Investigation, Writing – original draft, Writing – review & editing. **Janet Lubega Kobusinge:** Investigation, Writing – original draft, Writing – review & editing. **Gertrude Abbo:** Investigation, Writing – original draft, Writing – review & editing. **Annet Mary Namusisi:** Investigation, Writing – original draft, Writing – review & editing. **Bridget Ainembabazi:** Investigation, Writing – original draft, Writing – review & editing. **Patrick Kwizera:** Investigation, Writing – original draft, Writing – review & editing. **Wilfred Opeli:** Investigation, Writing – original draft, Writing – review & editing. **Winnie Agwang:** Investigation, Writing – original draft, Writing – review & editing. **Esther Nabatta:** Investigation, Writing – original draft, Writing – review & editing. **Tracy Maureen Rutogire:** Investigation, Writing – original draft, Writing – review & editing. **Ritah Namusoosa:** Investigation, Writing – original draft, Writing – review & editing. **Samuel Lugwana:** Investigation, Writing – original draft, Writing – review & editing. **Lilian Bulage:** Investigation, Writing – original draft, Writing – review & editing, Supervision. **Benon Kwesiga:** Conceptualization, Methodology, Resources, Supervision, Writing – review & editing. **Samuel Gidudu:** Conceptualization, Methodology, Resources, Supervision, Writing – review & editing. **Alex Riolexus Ario:** Conceptualization, Funding acquisition, Investigation, Methodology, Resources, Validation, Writing – review & editing.

## Declaration of competing interest

The authors have no competing interests to declare.
